# Heart Rate Estimated from Body Movements at Six Degrees of Freedom by Convolutional Neural Networks

**DOI:** 10.3390/s18051392

**Published:** 2018-05-01

**Authors:** Hyunwoo Lee, Mincheol Whang

**Affiliations:** 1 Department of Emotion Engineering, University of Sangmyung, Seoul 03016, Korea; lhw4846@naver.com; 2 Department of Intelligence Informatics Engineering, University of Sangmyung, Seoul 03016, Korea

**Keywords:** accelerometer, gyroscope, heart rate measurement, seismocardiography (SCG), wearable device, convolutional neural networks (CNNs)

## Abstract

Cardiac activity has been monitored continuously in daily life by virtue of advanced medical instruments with microelectromechanical system (MEMS) technology. Seismocardiography (SCG) has been considered to be free from the burden of measurement for cardiac activity, but it has been limited in its application in daily life. The most important issues regarding SCG are to overcome the limitations of motion artifacts due to the sensitivity of motion sensor. Although novel adaptive filters for noise cancellation have been developed, they depend on the researcher’s subjective decision. Convolutional neural networks (CNNs) can extract significant features from data automatically without a researcher’s subjective decision, so that signal processing has been recently replaced as CNNs. Thus, this study aimed to develop a novel method to enhance heart rate estimation from thoracic movement by CNNs. Thoracic movement was measured by six-axis accelerometer and gyroscope signals using a wearable sensor that can be worn by simply clipping on clothes. The dataset was collected from 30 participants (15 males, 15 females) using 12 measurement conditions according to two physical conditions (i.e., relaxed and aroused conditions), three body postures (i.e., sitting, standing, and supine), and six movement speeds (i.e., 3.2, 4.5, 5.8, 6.4, 8.5, and 10.3 km/h). The motion data (i.e., six-axis accelerometer and gyroscope) and heart rate (i.e., electrocardiogram (ECG)) were determined as the input data and labels in the dataset, respectively. The CNN model was developed based on VGG Net and optimized by testing according to network depth and data augmentation. The ensemble network of the VGG-16 without data augmentation and the VGG-19 with data augmentation was determined as optimal architecture for generalization. As a result, the proposed method showed higher accuracy than the previous SCG method using signal processing in most measurement conditions. The three main contributions are as follows: (1) the CNN model enhanced heart rate estimation with the benefits of automatic feature extraction from the data; (2) the proposed method was compared with the previous SCG method using signal processing; (3) the method was tested in 12 measurement conditions related to daily motion for a more practical application.

## 1. Introduction

Cardiac activity has been monitored continuously in daily life by virtue of advanced medical instruments with microelectromechanical systems (MEMS) technology. Seismocardiography (SCG) is one of the vital components that allows the possibility of this monitoring system. SCG is noninvasively measured from thoracic movements caused by both contraction of the heart and ejection of the blood from ventricles into the vasculature using motion sensors embedded in a wearable device [1]. SCG has less of a measurement burden and is more comfortable than traditional methods, such as electrocardiography (ECG) or photoplethysmography (PPG).

Despite the potential of SCG, it has still presented limited ability to be applied in daily life. The most important issues regarding SCG are to overcome the limitations of the measurement conditions according to measurement location, axis selection, and motion artifacts [2]. The limitations of measurement location and axis selection have been steadily improved in recent studies. First, the measurement location is related to the shape, amplitude, and clinical characteristics of the signal, so that initial SCG measurement systems have been developed to be forcibly contacted and fixed on the left side of the chest [3,4,5,6,7]. Recently, ref. [8] a portable and wearable apparatus has been developed that is capable of measuring SCG by simply being clipped onto clothing. Second, the axis of the signal should be selected by considering the purpose of the measurement because the characteristics of the signal depend on the axis selection as well as the measurement location. The initial SCG studies only focused on the *z*-axis of the accelerometer, but recent studies have explored the clinical interpretation and integration for the tri-axis of the accelerometer [4,9,10,11], the tri-axis of the gyroscope [12,13,14,15], and the six-axis of the accelerometer and gyroscope [8].

However, the limitations of the motion artifacts still need to be improved for application in daily life. Because SCG is measured by capturing only thoracic movement associated with heartbeat from body movement, it is important to develop the signal processing to reduce the motion artifacts. Pandia et al. [10] proposed that the Savitzky–Golay filter has the advantage of denoising by preserving higher order moments around inflection points. They demonstrated enhanced detection rate of peaks of SCG in walking at normal speed. Di Rienzo et al. [4] developed noise cancellation using ensemble averaging of repeatedly measured accelerometer signals and proved it by beat-by-beat assessment of cardiac mechanics (e.g., pre-ejection period (PEP)) in three measurement conditions according to supine, standing, and spontaneous behavior. Yang and Tavassolian [16] proposed normalized least mean square (NLMS) adaptive filter using the *y*-axis of the accelerometer as a reference signal to cancel the motion artifacts. They tested their method by detection rate of peaks of SCG in four measurement conditions according to random behavior, fast walking, eating, and drinking. Javaid et al. [17] developed a novel method to assess left ventricular health using the *z*-axis of the accelerometer by empirical mode decomposition (EMD) and feature-tracking algorithms. They proved it by PEP in three measurement conditions according to walking at normal and fast (1.34–1.45 m/s) speeds and at a brisk pace. Lee et al. [8] developed an enhanced method to estimate heart rate using ensemble averaging of the six-axis of the accelerometer and gyroscope. They proved it in four measurement conditions according to physical conditions (e.g., relaxed and aroused conditions) and body postures (e.g., standing and sitting). Although novel adaptive filters for noise cancellation have been developed, they depend on fewer (fewer than four) measurement conditions and the researcher’s subjective decision. Thus, it is necessary to develop better robust noise cancellation without the subjective decision and to demonstrate them in more measurement conditions.

Since convolutional neural networks (CNNs) have been developed by [18] in 1989, CNNs have demonstrated superior performance at visual classification problems [19]. Furthermore, the principles of CNNs have been explored to understand how CNNs achieve excellent performance by visualization technologies [20,21]. They reported that the invariant and discriminative features were automatically extracted from raw data by convolution layers. This result showed that the data-based features outperform the hand-created features determined by the researcher’s decision. CNNs have recently achieved state-of-the-art performance at time-series problems (e.g., natural language [22], sound [23], and human motion [24]) as well as the visual classification problems. Traditional methods for the time-series problems have employed a digital filter to remove the noise and to extract the significant features. The convolution has been operated to suppress some aspect of signal frequency in the digital filter process [25]. Thus, the convolution layers of CNNs can replace the digital filter and can automatically extract significant features from raw time-series data. This study hypothesized that the significant features relevant to cardiac activity can be extracted from the motion data better by CNNs than by previous methods based on signal processing determined by the researcher’s subjective decision.

This study was conducted to develop a novel method to enhance the heart rate estimation from thoracic movement by CNNs. Thoracic movement was measured by six-axis accelerometer and gyroscope signals using a wearable sensor that can be worn by simply clipping on clothes. The dataset for training the CNN model was collected from 30 persons in 12 measurement conditions according to body postures, physical conditions, and movement speeds. The CNN model was developed based on VGG Net [26] and optimized by testing according to network depth and data augmentation. It was evaluated by calculating accuracy from ECG measured as ground truth and was compared with the previous SCG method using signal processing. The contributions of this study can be summarized as follows: (1) the CNN model enhanced heart rate estimation with the benefits of automatic feature extraction from the data; (2) the proposed method was compared with the previous SCG method using signal processing; (3) the method was tested in 12 measurement conditions related to daily motion for a more practical application.

## 2. Dataset

### 2.1. Experiment

This study was an experiment designed to collect a dataset for training and evaluating the CNN model. The participants consisted of 30 persons (15 males, 15 females) aged 27.7 ± 3.3 years. They had no medical history related to cardiovascular disease and were healthy enough to perform physical exercise. All participants were instructed to have a full rest of sleep and were asked to abstain from caffeine, alcohol, and cigarettes before the experiments. They provided written informed consent before the experiment and were paid as an incentive after the experiment.

**Figure 1:**
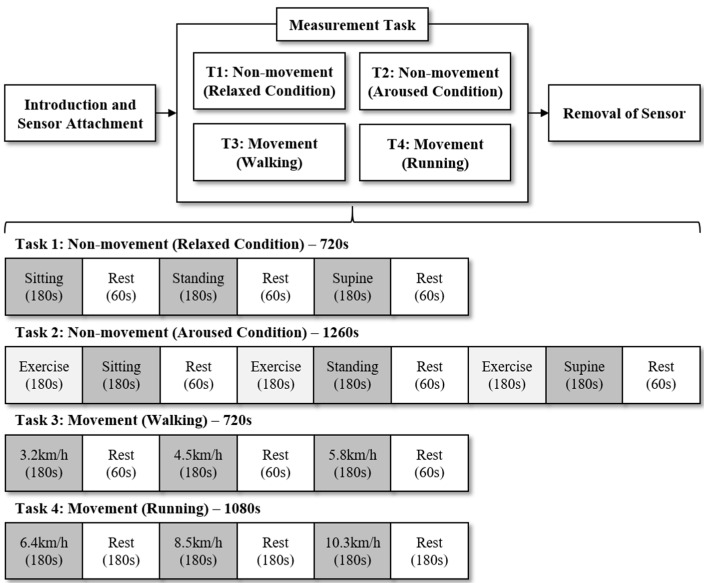
Experimental procedure according to two physical conditions, three body postures, and six movement speeds. The experiment lasted for a total of 63 min and sufficient rest was given between each task.

One of the challenges for SCG is that the accelerometer signals to determine SCG is sensitive to body postures and motion artifacts. In addition, it is necessary to test SCG for a wide range of heart rates to ensure clinical relevance. Thus, this study verified the method in 12 measurement conditions according to body postures, physical conditions, and movement speeds by considering the mentioned challenges, as shown in Figure 1. First, in six measurement conditions according to body postures and physical conditions, all participants were asked to maintain three body postures (i.e., sitting, standing, and supine) in two physical conditions (i.e., relaxed and aroused conditions). To evoke the physically relaxed condition, the participants closed their eyes and maintained the body postures for 3 min. To evoke the physically aroused condition, they exercised and maintained the body postures for 3 min. The exercise was to make the heart beat faster by running on the treadmill at speed of 8.5 km/h for 3 min. Second, in six measurement conditions according to movement speeds, they were requested to walk and to run at six speeds (i.e., 3.2, 4.5, 5.8, 6.4, 8.5, and 10.3 km/h) for 3 min, respectively. The experiment lasted for a total of 63 min and sufficient rest was given between each task. The participants were given longer rest times after exercise tasks than after nonexercise tasks by taking into account the time required for cardiac activity to be restored to a steady condition. This protocol was approved by the Institutional Review Board of the Sangmyung University, Seoul, South Korea (BE2016-14).

**Figure 2:**
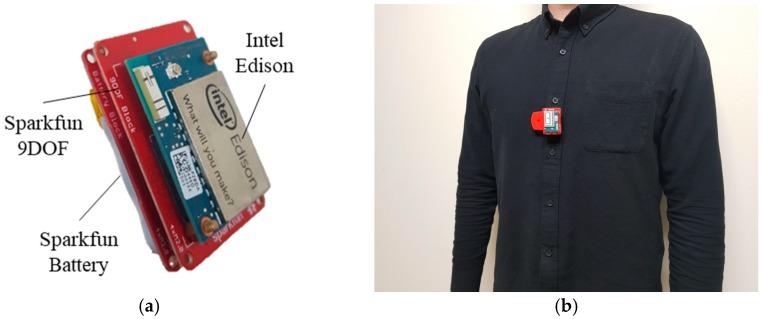
Overview of the apparatus. (**a**) Packaging of the Intel Edison, Sparkfun 9DOF, and Sparkfun Battery; (**b**) Appearance of the apparatus when worn by participant.

This study employed a portable and wearable apparatus, which had been developed in our previous study [8], to measure thoracic movement using the MEMS accelerometer and gyroscope sensors. The apparatus was worn by the participants by simply clipping it onto their clothing around the left side of the chest, as shown in Figure 2. The tri-axis accelerometer and tri-axis gyroscope signals were measured at sampling rates within the range of around 100 and 200 Hz using the apparatus. ECG was simultaneously measured using an ECG measurement system with Lead-I. The system consisted of an ECG 100C amplifier system and a MP150 data acquisition system (BIOPAC Systems Inc., Goleta, CA, USA). ECG was measured at a sampling rate of 512 Hz and served as a ground truth for the evaluation of SCG. In order to reduce noise while measuring ECG, the participants were asked to minimize the movement of their arms during the experiment.

### 2.2. Formatting

**Figure 3:**
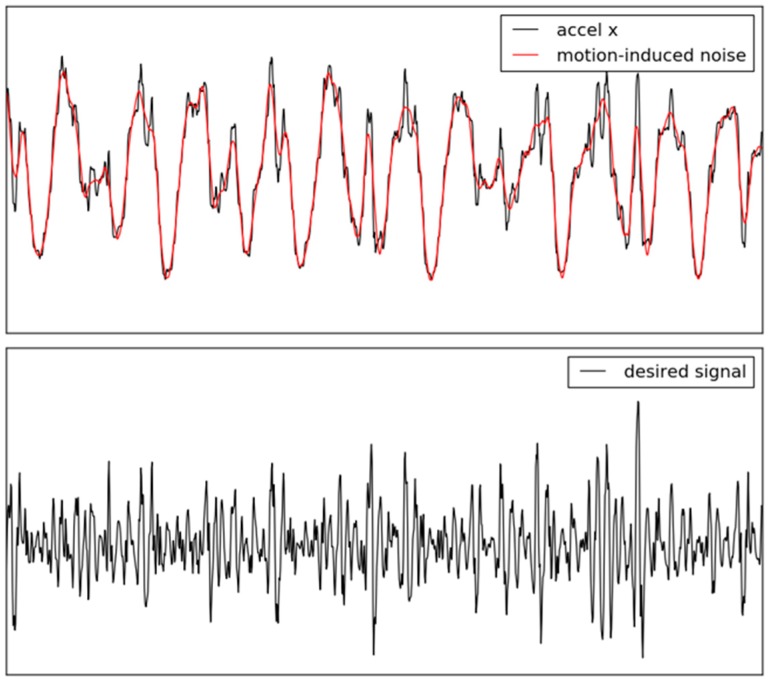
Motion artifacts reduction by the Savitzky–Golay filter.

It is necessary to collect a large-scale dataset to train CNNs well. Thus, this study augmented the dataset by dividing the motion data and ECG with a sliding window (window size = 5 s, interval size = 1 s). Then, the motion data and ECG were preprocessed to transform them into a data structure for CNNs. The six-axis motion data was measured from the accelerometer and gyroscope at a sampling rate within the range of around 100 and 200 Hz, as described in Section 2.1. The motion data was interpolated as the sampling rate of 256 Hz by cubic spline interpolation [27] because CNNs take fixed-size data as input data. The motion-induced noise was estimated by the Savitzky–Golay filter with the order of 2 and window size of 31 samples [28] and was subtracted from each accelerometer or gyroscope signal to remove large-range motion, as shown in Figure 3. This method preserves higher order moments around inflection points and overcomes the limit of the simple digital filter [10]. The accelerometer and gyroscope signals were normalized to have an average of zero and a variance of one. ECG was measured at the sampling rate of 512 Hz to serve as ground truth. The R-R Intervals (RRIs) were calculated from ECG by the QRS detection algorithm, which was implemented by Pan and Tompkins to detect the R peaks [29]. The heart rate was calculated from the average of the RRIs. Finally, the motion data and heart rate were determined as the input data and labels in the dataset, respectively.

The total number of the dataset is 58,561 samples. If the heart rate is lower than 60 or is higher than 200, this sample was not included in the dataset because ECG might have been measured incorrectly. The dataset was shuffled and then divided into training data (70%), validation data (10%), and test data (20%) for the cross-validation method.

## 3. Convolutional Neural Networks

### 3.1. Baseline Architecture

**Figure 4:**
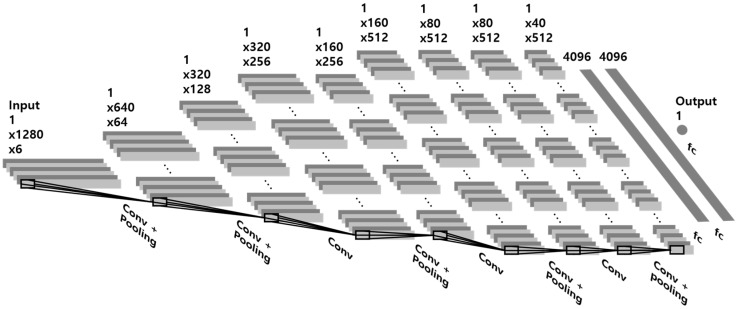
Baseline network architecture based on VGG-11.

This study proposed a network architecture with CNN for estimating the heart rate from motion data on the chest, as shown in Figure 4. The baseline network architecture with eight convolutional layers and three fully connected layers was developed based on VGG-11 [26] and tuned to apply the motion data. There are three approaches to apply the motion data. First, although the input format of CNNs were originally regarded as a square structure proposed for image data, it was reshaped as a rectangle structure (1 × 1280 × 6) in this study. Second, this study employed the convolution (1 × 3) and pooling (1 × 2) operations with the 1-D rectangle shape instead of the 2-D square shape. Third, this network had one output node to solve a regression problem because it estimated continuous data (i.e., heart rate). In the next section, several network architectures are examined according to additional convolutional layers (e.g., VGG-13, VGG-16, and VGG-19) and data augmentation (e.g., permutation, jittering, and scaling).

There are several hyperparameters to be determined for training and optimization: activation functions, loss functions, optimizer, learning rate, accuracy, etc. Rectified units [30] are employed as the activation function to reduce the vanishing gradient problem. The optimization was performed by the L2-loss function and Adam optimizer [31] with a learning rate of 0.0001. Additionally, the dropout [32] with a probability of 0.5 was involved in each fully connected layer to avoid overfitting. The network was initialized by Xavier initialization [33] and was trained with 128-sized minibatches. The accuracy was calculated to evaluate the performance of network as

(1) $$Accuracy=\left(1-\frac{1}{n}\overset{n}{\underset{i=0}{\sum }}\frac{\left|\hat{y_{i}}-y_{i}\right|}{y_{i}}\right)\times 100$$

where y is true label, $\hat{y}$ is predicted label, and n is number of samples in the dataset.

The network architecture was tested according to network depth and data augmentation. The network was implemented with TensorFlow [34], an open source deep-learning library, using a computer equipped with 3.6 GHz quad-core processors and 4 NVIDIA GeForce GTX 1080 GPUs. The dataset used in the experiments consisted of 40,986 training data, 5858 validation data, and 11,717 test data.

### 3.2. Effects of CNN Depth

**Figure 5:**
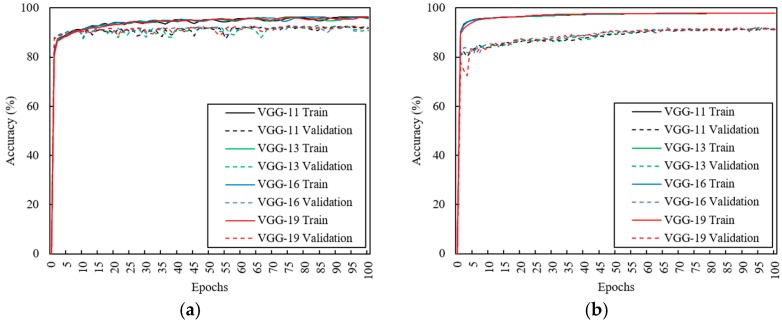
Learning curves of VGG-11, VGG-13, VGG-16, and VGG-19 for training and validation data according to 100 training epochs. (**a**) Raw dataset; (**b**) Augmented dataset.

This study investigated the effect of network depth on its accuracy in our dataset. Note that depending on the network depth, the invariance and discriminative feature maps can be represented in a higher layer [26]. However, since an excessively deep network is difficult to generalize because of overfitting, an appropriate depth needed to be determined for our dataset. There were four network architectures according to the depth: VGG-11, VGG-13, VGG-16, and VGG-19. Each network was tuned to apply the motion data and to estimate the heart rate as described above (Baseline architecture). Then, it was trained for 100 epochs with training data, including all measurement conditions (see Figure 5a). The model’s parameters were saved when the validation cost was lowest.

### 3.3. Effects of Data Augmentation

**Figure 6:**
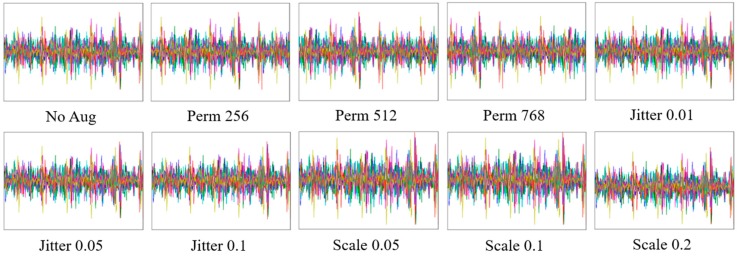
Data augmentation consisting of permutation, jittering, and scaling.

Although deeper networks can represent the invariance and discriminative feature maps, it is necessary for a large-scale dataset to train well without overfitting. However, it was difficult to collect a large-scale dataset in our experiments which involved human subjects. Thus, this study employed data augmentation to create a large-scale dataset and examined the effect of data augmentation on the network’s accuracy in our dataset. For data augmentation, the domain knowledge should be considered to preserve the labels after transformations. For example, image processing methods such as jittering, scaling, copping, distorting, or rotating are well known as the data augmentation on CNNs studies for vision. This study employed data augmentation, which was developed to generate new motion data from existing motion data, such as permutation, jittering, and scaling, as shown in Figure 6 [35]. Permutation creates new data by moving the temporal location as

(2) $$Perm\left(x,\alpha \right)=\{\begin{array}{c} x_{n-\alpha +i},\;i<\alpha \\ x_{i-\alpha },\;i\geq \alpha \end{array}$$

where x is motion data, n is length of data, and α is the window size for moving the temporal location. It can represent the invariant features for temporal location. Jittering distorts the data by adding the noise with a gaussian distribution as

(3) $$gaussian\left(x,m,\alpha \right)=\frac{1}{\sqrt{2\pi }\sigma }\times e^{-\frac{{\left(x-m\right)}^{2}}{2\sigma }}$$

where m is a mean of distribution and σ is a standard deviation of distribution.

(4) Jittering(x,α)=x+gaussian(x,0,α)

where x is motion data and α is a standard deviation of noise distribution. Scaling increases or decreases the amplitude of data by multiplying random value as

(5) Scaling(x,α)=x×(1+α)

where x is motion data and α is a scaling ratio. Jittering and scaling can represent the invariant features for noise. Each network was trained for 100 epochs with augmented training data and were saved when their validation cost was lowest, respectively (see Figure 5b).

### 3.4. Evaluation of Structural Risks

The structural risk of network architectures was defined as the instability of the method using the A-Test proposed in [36], based on the multiple use of *z*-fold validation. Because this study solved the regression problem, the regression error $\tau_{n,z}$ of a network n was defined as mean absolute error (MAE). The metric $\hat{\tau_{n}}$ for estimating the structural risk of a network n was calculated by the average of the regression error $\tau_{n,z}$ as

(6) $$\hat{\tau_{n}}=\frac{\sum_{z=2}^{Z_{max}}\tau_{n,z}}{Z_{max}-1}$$

where $Z_{max}$ is determined as 10-fold in this study. Each fold group was divided to distribute heart rates uniformly. The low value of $\hat{\tau_{n}}$ corresponds to a low structural risk, and the minimum value of $\tau_{n}$ indicates the potential of the network to achieve better performance with a larger dataset.

### 3.5. Optimal Architecture

Table 1 presents the accuracy of heart rate estimation according to the CNN depth and the data augmentation. The VGG-16 without data augmentation shows the highest accuracy and lowest structural risk in nonmovement conditions, whereas the VGG-19 with data augmentation shows the highest accuracy and lowest structural risk in movement conditions. As a generalization, heart rate estimation should be accurate for all measurement conditions, with or without movement. Thus, this study determined the ensemble network of the VGG-16 without data augmentation and the VGG-19 with data augmentation as optimal architecture as

(7) $$HR_{ensemble}=\frac{\left(HR_{vgg16\left(noaug\right)}+HR_{vgg19\left(aug\right)}\right)}{2}$$

where $HR_{vgg16\left(noaug\right)}$ is heart rate estimated by the VGG-16 without data augmentation, $HR_{vgg19\left(aug\right)}$ is heart rate estimated by the VGG-19 with data augmentation, and $HR_{ensemble}$ is heart rate finally estimated by the ensemble network.

## 4. Results

The three CNN models (i.e., VGG-16 (No Aug), VGG-19 (Aug), and ensemble network) were evaluated by comparing them with the heart rate of ECG by mean absolute error (MAE), standard deviation of absolute error (SDAE), root mean squared error (RMSE), and Pearson’s correlation coefficients (CC). In addition, they were compared to each other by the Bland-Altman plot [37], which is represented by assigning the mean (*x*-axis) and difference (*y*-axis) between the two measurements with the 95% limits of an agreement calculated by mean difference and the ±1.96 standard deviation of the difference. Finally, the ensemble network, which was determined to be the optimal architecture for generalization, was compared with the previous SCG method [8], which employed ensemble averaging of the six-axis of the accelerometer and gyroscope using signal processing.

### 4.1. Estimation of Heart Rate in Relaxed Condition

The heart rates were estimated from the six-axis of the accelerometer and gyroscope by the VGG-16 without data augmentation (VGG-16 (No Aug)), the VGG-19 with data augmentation (VGG-19 (Aug)), and their ensemble network, respectively. The heart rates for sitting, standing, and supine postures in relaxed condition were evaluated as shown in Table 2. The errors for sitting posture were lower with the VGG-16 (No Aug) than the other networks (MAE = 1.92, SDAE = 2.40, RMSE = 3.08), but the correlation coefficient was highest with the ensemble network (CC = 0.960). The VGG-16 (No Aug) showed the lowest errors for standing (MAE = 1.72, SDAE = 1.69, RMSE = 2.40, CC = 0.984) and supine (MAE = 1.67, SDAE = 2.60, RMSE = 3.09, CC = 0.982) postures, respectively.

**Figure 7:**
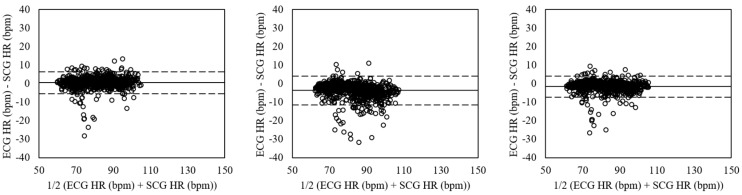

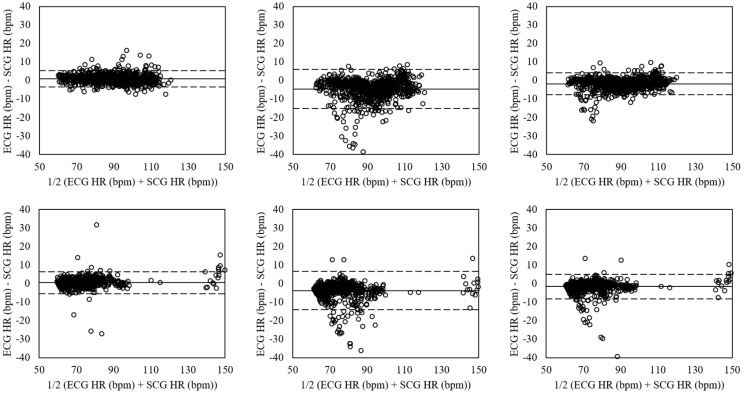
Bland-Altman plots of heart rates estimated from SCG and ECG by CNNs in relaxed condition for sitting (**Left**), standing (**Mid**), and supine (**Right**) postures based on the VGG-16 without data augmentation (**Top**), VGG-19 with data augmentation (**Mid**), and ensemble network (**Down**). The lines are the mean errors and 95% LOA.

The Bland-Altman plots of heart rates evaluated for sitting, standing, and supine postures in relaxed condition by each estimation method are shown in Figure 7. The mean errors for sitting posture (left plots) were 0.57 with 95% LOA in −5.36–6.50 (VGG-16 (No Aug)), −3.65 with 95% LOA in −11.43–4.13 (VGG-19 (Aug)), and −1.54 with 95% LOA in −7.23–4.15 (ensemble network). The standing posture (mid plots) showed the mean errors of 0.83 with 95% LOA in −3.60–5.25 (VGG-16 (No Aug)), −4.55 with 95% LOA in −15.09–5.98 (VGG-19 (Aug)), and −1.86 with 95% LOA in −7.79–4.07 (ensemble network). The supine posture (right plots) had mean errors of 0.45 with 95% LOA in −5.54–6.44 (VGG-16 (No Aug)), −3.76 with 95% LOA in −14.09–6.57 (VGG-19 (Aug)), and −1.66 with 95% LOA in −8.36–5.05 (ensemble network).

### 4.2. Estimation of Heart Rate in Aroused Condition

Table 3 presents the heart rates evaluated for sitting, standing, and supine postures in aroused condition. The errors for sitting posture were lower with the VGG-16 (No Aug) than the other networks (MAE = 2.23, SDAE = 3.92, RMSE = 4.51, CC = 0.976). Similarly, the errors of the VGG-16 (No Aug) were lower than the errors of the other networks for standing (MAE = 2.34, SDAE = 3.47, RMSE = 4.19, CC = 0.975) and supine (MAE = 1.51, SDAE = 1.57, RMSE = 2.18, CC = 0.992) postures.

**Figure 8:**
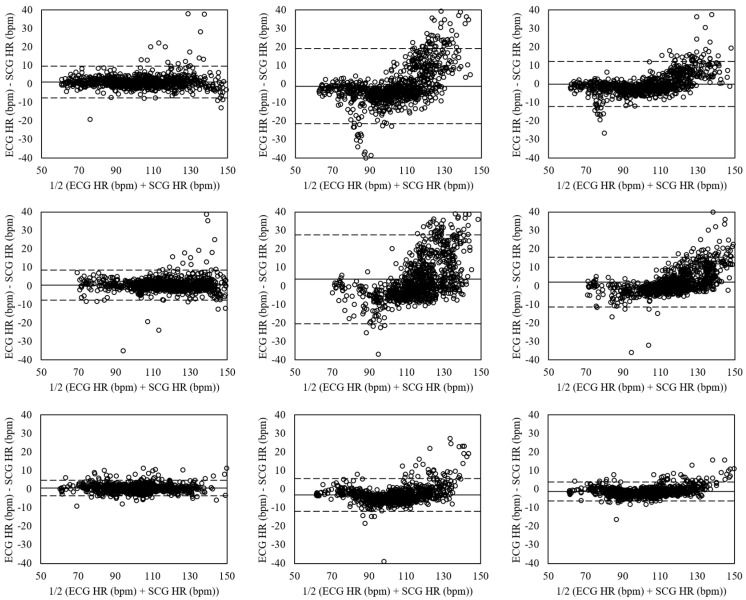
Bland-Altman plots of heart rates estimated from SCG and ECG by CNNs in aroused condition for sitting (**Left**), standing (**Mid**), and supine (**Right**) postures based on the VGG-16 without data augmentation (**Top**), VGG-19 with data augmentation (**Mid**), and ensemble network (**Down**). The lines are the mean errors and 95% LOA.

Figure 8 shows the Bland-Altman plots of heart rates evaluated for sitting, standing, and supine postures in aroused condition by the ensemble averaging and the VGG-19. The sitting posture (left plots) presented the mean errors of 1.13 with 95% LOA in −7.43–9.69 (VGG-16 (No Aug)), −1.12 with 95% LOA in −21.47–19.22 (VGG-19 (Aug)), and 0.00 with 95% LOA in −12.18–12.19 (ensemble network). The mean errors of standing posture (mid plot) were 0.46 with 95% LOA in −7.71–8.62 (VGG-16 (No Aug)), 3.62 with 95% LOA in −20.35–27.59 (VGG-19 (Aug)), and 2.04 with 95% LOA in −11.41–15.48 (ensemble network). The supine posture (right plots) had the mean errors of 0.48 with 95% LOA in −3.69–4.65 (VGG-16 (No Aug)), −3.16 with 95% LOA in −12.03–5.71 (VGG-19 (Aug)), and −1.34 with 95% LOA in −6.41–3.73 (ensemble network).

### 4.3. Estimation of Heart Rate for Walking

Table 4 shows the heart rates evaluated for walking at movement speeds of 3.2, 4.5, and 5.8 km/h. The errors at movement speed of 3.2 km/h were lower with the ensemble network than the VGG-16 (No Aug) and VGG-19 (Aug) (MAE = 5.03, SDAE = 5.29, RMSE = 7.30, CC = 0.930). Similarly, the errors of the ensemble network were lower than the errors of the other network at movement speeds of 4.5 km/h (MAE = 4.26, SDAE = 5.35, RMSE = 6.84, CC = 0.935). On the other hand, the errors at movement speeds of 5.8 km/h were lowest with the VGG-19 (Aug) (MAE = 4.74, SDAE = 5.30, RMSE = 7.11, CC = 0.935).

**Figure 9:**
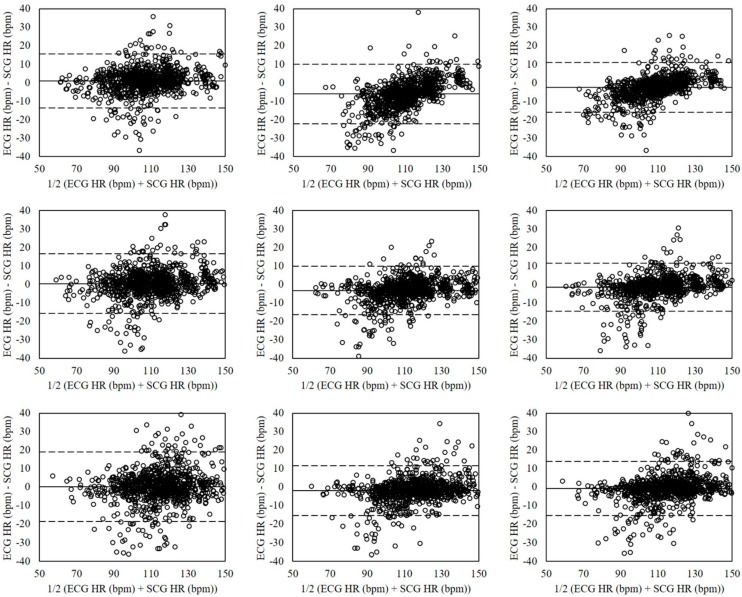
Bland-Altman plots of heart rates estimated from SCG and ECG by CNNs for walking at movement speeds of 3.2 (**Left**), 4.5 (**Mid**), and 5.8 (**Right**) km/h based on the VGG-16 without data augmentation (**Top**), VGG-19 with data augmentation (**Mid**), and ensemble network (**Down**). The lines are the mean errors and 95% LOA.

The Bland-Altman plots of heart rates evaluated for walking at movement speeds of 3.2, 4.5, and 5.8 km/h by the ensemble averaging and the VGG-19 are shown in Figure 9. The movement speed of 3.2 km/h (left plots) had the mean errors of 1.00 with 95% LOA in −13.62–15.62 (VGG-16 (No Aug)), −6.10 with 95% LOA in −22.27–10.07 (VGG-19 (Aug)), and −2.55 with 95% LOA in −15.96–10.87 (ensemble network). The mean errors at movement speed of 4.5 km/h (mid plots) were 0.35 with 95% LOA in −15.76–16.47 (VGG-16 (No Aug)), −3.35 with 95% LOA in −16.47–9.76 (VGG-19 (Aug)), and −1.50 with 95% LOA in −14.58–11.58 (ensemble network). The movement speed of 5.8 km/h (right plots) had the mean errors of 0.20 with 95% LOA in −18.65–19.04 (VGG-16 (No Aug)), −1.82 with 95% LOA in −15.29–11.65 (VGG-19 (Aug)), and −0.81 with 95% LOA in −15.47–13.85 (ensemble network).

### 4.4. Estimation of Heart Rate for Running

The heart rates for running at movement speeds of 6.4, 8.5, and 10.3 km/h were evaluated as shown in Table 5. The errors at movement speed of 6.4 km/h were lower with the VGG-19 (Aug) than other the other networks (MAE = 5.24, SDAE = 6.72, RMSE = 8.52, CC = 0.917). Similarly, the errors of the VGG-19 (Aug) were lower than the errors of the other networks at movement speeds of 8.5 (MAE = 5.21, SDAE = 7.14, RMSE = 8.84, CC = 0.924) and 10.3 (MAE = 5.49, SDAE = 7.97, RMSE = 9.68, CC = 0.908) km/h.

**Figure 10:**
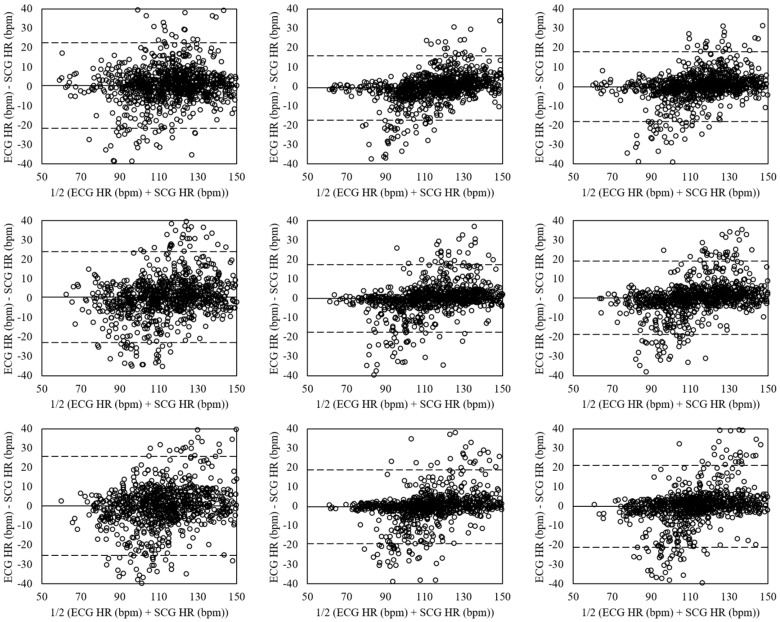
Bland-Altman plots of heart rates estimated from SCG and ECG by CNNs for running at movement speeds of 6.4 (**Left**), 8.5 (**Mid**), and 10.3 (**Right**) km/h based on the VGG-16 without data augmentation (**Top**), VGG-19 with data augmentation (**Mid**), and ensemble network (**Down**). The lines are the mean errors and 95% LOA.

Figure 10 shows the Bland-Altman plots of the heart rates estimated for running at movement speeds of 6.4, 8.5, and 10.3 km/h. The mean errors at movement speed of 6.4 km/h (left plots) were 0.45 with 95% LOA in −21.59–22.50 (VGG-16 (No Aug)), −0.73 with 95% LOA in −17.37–15.92 (VGG-19 (Aug)), and −0.14 with 95% LOA in −18.12–17.85 (ensemble network). The mean errors at movement speed of 8.5 km/h (mid plots) were 0.46 with 95% LOA in −22.98–23.90 (VGG-16 (No Aug)), −0.13 with 95% LOA in −17.46–17.19 (VGG-19 (Aug)), and 0.16 with 95% LOA in −18.80–19.13 (ensemble network). The movement speed of 10.3 km/h had the mean errors of 0.23 with 95% LOA in −25.36–25.83 (VGG-16 (No Aug)), −0.30 with 95% LOA in −19.27–18.67 (VGG-19 (Aug)), and −0.03 with 95% LOA in −21.05–20.98 (ensemble network).

### 4.5. Comparison with Previous SCG Method Using Signal Processing

Table 6 shows the heart rates evaluated by signal processing (previous method) and CNN (proposed method) in 12 measurement conditions. The errors of signal processing were lower than the errors of CNN in four measurement conditions: standing posture in relaxed condition (MAE = 2.00, SDAE = 2.33, RMSE = 3.04, CC = 0.975); sitting posture in aroused condition (MAE = 1.93, SDAE = 3.81, RMSE = 4.22, CC = 0.973); standing posture in aroused condition (MAE = 2.46, SDAE = 2.59, RMSE = 3.54, CC = 0.981); supine posture in aroused condition (MAE = 1.64, SDAE = 2.53, RMSE = 2.98, CC = 0.986). On the other hand, the errors of CNN were lower than the errors of signal processing in eight measurement conditions: sitting posture in relaxed condition (MAE = 2.07, SDAE = 2.56, RMSE = 3.29, CC = 0.960); supine posture in relaxed condition (MAE = 2.17, SDAE = 3.12, RMSE = 3.80, CC = 0.977); walking at movement speed of 3.2 km/h (MAE = 5.03, SDAE = 5.29, RMSE = 7.30, CC = 0.930); walking at movement speed of 4.5 km/h (MAE = 4.26, SDAE = 5.35, RMSE = 6.84, CC = 0.935); walking at movement speed of 5.8 km/h (MAE = 4.76, SDAE = 5.83, RMSE = 7.52, CC = 0.922); running at movement speed of 6.4 km/h (MAE = 5.43, SDAE = 7.40, RMSE = 9.17, CC = 0.899); running at movement speed of 8.5 km/h (MAE = 5.94, SDAE = 7.64, RMSE = 9.67, CC = 0.908); running at movement speed of 10.3 km/h (MAE = 6.38, SDAE = 8.61, RMSE = 10.72, CC = 0.886). Overall, the CNN showed better performance than the signal processing in most measurement conditions, especially in movement conditions.

## 5. Discussion

This study developed the CNN model to replace traditional signal processing and to enhance heart rate estimation. The networks were evaluated on effects of CNN depth and data augmentation according to 12 measurement conditions. The VGG-16 without data augmentation was better than the other networks in nonmovement conditions, whereas the VGG-19 with data augmentation was better than the other networks in movement conditions. Their ensemble network was determined as the optimal architecture for generalization in this study.

Overall, this study has drawn six significant findings. First, for supine posture in the relaxed condition, the signal processing-based method showed high error (MAE = 18.05, SDAE = 17.27, RMSE = 24.78) and low correlation (CC = −0.084), as shown in Table 6. It indicated that the motion data did not sufficiently reflect the thoracic movement and that the apparatus may not be in close contact with the body for supine posture. Note that, nevertheless, the CNN-based method showed low error (MAE = 2.17, SDAE = 3.12, RMSE = 3.80) and high correlation (CC = 0.977). It indicated that the CNN-based method can extract features that cannot be extracted by signal processing. Thus, the CNN-based method can increase the possibility of heart rate estimation in daily life when the apparatus is not fixed.

Second, the accuracy was lower as the movement speed increased in most movement conditions. Faster movement speed leads to larger motions and induces the motion data to include more noise. However, the accuracy for walking at movement speed of 3.2 km/h was higher than the one for walking at movement speeds of 4.5 and 5.8 km/h. It may be interpreted that the motion for walking at movement speed of 3.2 km/h causes more noise to the frequency components associated with the heartbeat. Note that the noise cancellation should be focused on the noise associated with the frequency components rather than the time components.

Third, before data augmentation, the average accuracy for all measurement conditions was higher with the deeper networks (e.g., VGG-16 or VGG-19) than the shallower networks (e.g., VGG-11 or VGG-13). However, the effect of the depth was different depending on whether the test data included movement conditions or not. The deeper networks were better than the shallower networks in nonmovement conditions as well as all measurement conditions, but the shallower networks were better than the deeper networks in movement conditions. The deeper networks can extract more invariant features than the shallower networks. However, it is necessary for a large-scale dataset to train the deeper network without overfitting. The motion data in movement conditions has more large variation than in nonmovement conditions, thus, it is difficult to train the deeper networks without overfitting. Note that in the results after data augmentation, the deeper networks were better than shallower networks in most nonmovement conditions as well as movement conditions. It indicated that the data augmentation, which allows for the extraction of invariant features for temporal location and noise, is important in training the network with the noisy motion data.

Fourth, data augmentation improved heart rate estimation in movement conditions, but not in nonmovement conditions. It can be interpreted that our network models are insufficient for extracting the invariant features from both the nonmovement and movement conditions. As a generalization, it is important not only to apply data augmentation and to optimize hyper parameters, but also to improve the network architectures. CNNs have been recently developed with a focus on structural improvements to increase the number of layers and to reduce the number of parameters [21,38,39]. If our network is structurally improved in the future, it is expected that the heart rate estimation will be improved in both nonmovement and movement conditions.

Fifth, the accuracy of heart rate estimation was higher with the single networks (i.e., VGG-16 (No Aug) and VGG-19 (Aug)) than with the ensemble network, but the single networks showed higher accuracy only in certain conditions. For example, although the accuracy of the VGG-16 (No Aug) was high in nonmovement conditions, it was low in movement conditions. On the other hand, the accuracy of the VGG-19 (Aug) was low in nonmovement conditions but high in movement conditions. These results indicate that single networks are difficult to employ for general-purpose applications. In order to be employed for general-purpose applications in daily life, reasonable performance should be ensured in most measurement conditions. Thus, this study suggests that the ensemble network is the optimal network architecture for general-purpose applications of heart rate estimation. However, the ensemble network is more complex and requires more capacity than a single network and traditional signal processing, thus making it difficult to load our method onto an embedded system. This study proposed solutions to develop the distillation method [40] to preserve the performance of an ensemble network by using single networks.

Sixth, the proposed method showed better performance than the previous method using signal processing in most measurement conditions, especially in movement conditions. However, the machine-learning methods have shown limited ability in unexpected conditions. Because daily conditions are so diverse that they cannot all be considered in the experiment, it is necessary to develop a method to improve performance even in unexpected conditions. This study proposed solutions to develop additional data augmentation for invariant features and to create virtual data in unexpected conditions by generative models, such as variational auto-encoder (VAE) [41] and generative adversarial networks (GANs) [42].

This study explored the issues regarding SCG in measurement conditions according to measurement location, axis selection, and motion artifacts. First, the possibility for a more comfortable measurement location was shown by the apparatus being simply worn on clothes. Second, the six-axis of the accelerometer and gyroscope were integrated to extract significant features related to thoracic movement. Third, the proposed method using CNN was demonstrated to better reduce motion artifacts than traditional signal processing and to estimate more accurately the heart rate in movement conditions. Consequently, our findings represent a significant step towards ensuring the enhanced development of SCG.

## 6. Conclusions

This study estimated the heart rate in 12 measurement conditions according to body postures, physical conditions, and movement speeds. The proposed method using CNNs was compared with the previous SCG method using traditional signal processing. As a result, the proposed method estimated a more accurate heart rate than traditional SCG methods by employing ensemble averaging of the six-axis of the accelerometer and gyroscope. Specifically, CNNs demonstrated the ability to overcome the motion artifacts problem for SCG by replacing traditional signal processing. The findings are a significant step towards ensuring the enhanced development of SCG. This study is expected to help more accurately estimate the heart rate by overcoming the motion artifacts problem and consequently improving the monitoring environment of wearer-comfortability devices in daily life.

## Figures and Tables

**Table 1:** Effects of CNN depth and data augmentation according to 12 measurement conditions and results of the A-Test for evaluating structural risks.

		No Aug				Aug				EN
		VGG-11	VGG-13	VGG-16	VGG-19	VGG-11	VGG-13	VGG-16	VGG-19	
Train	All	97.46	97.33	97.46	97.19	95.32	95.08	95.27	95.33	-
Test	Sit (R)	97.20	97.14	**97.57**	97.41	93.65	**94.22**	93.45	95.16	**96.37**
	Stand (R)	97.67	97.73	**98.06**	97.94	93.86	**94.34**	93.83	94.06	**96.06**
	Sup (R)	97.30	97.31	97.79	**97.83**	93.29	93.07	92.77	**94.25**	**96.02**
	Sit (A)	97.75	97.67	97.92	**97.95**	93.89	94.54	**94.95**	92.74	**95.33**
	Stand (A)	97.55	97.53	**98.01**	97.71	94.11	94.98	**95.12**	93.11	**95.56**
	Sup (A)	98.16	98.09	**98.49**	98.21	95.15	95.34	**95.82**	95.55	**97.02**
	Walk (3.2)	95.19	**95.20**	95.11	94.79	92.45	91.83	92.07	91.90	**93.51**
	Walk (4.5)	94.49	94.52	**94.72**	94.54	94.37	92.78	94.05	**94.88**	**94.80**
	Walk (5.8)	**94.54**	94.34	94.21	94.15	95.4	94.26	95.01	**95.60**	**94.91**
	Run (6.4)	**93.54**	93.31	93.29	93.36	94.69	93.61	94.47	**95.03**	**94.16**
	Run (8.5)	**92.83**	92.35	92.57	92.17	95.09	94.98	94.80	**95.11**	**93.84**
	Run (10.3)	**92.48**	92.20	92.05	91.70	94.62	95.18	94.85	**94.93**	**93.49**
	All	95.72	95.62	**95.82**	95.65	94.21	94.09	94.27	**94.36**	**95.09**
A-Test	$\hat{\tau_{n}}$	7.95	7.83	7.61	**7.57**	8.40	8.06	7.88	**7.81**	**6.98**
	$\min\tau_{n}$	3.11	2.97	**2.43**	2.44	5.21	5.10	**4.67**	4.82	**2.85**

No Aug = No Augmentation; Aug = Augmentation; EN = Ensemble Network. Bolded numbers are the highest accuracy according to 12 measurement conditions in No Aug and Aug models, respectively. Red numbers are the highest accuracy according to 12 measurement conditions in all models. Optimal architecture is the ensemble network of VGG-16 without data augmentation and VGG-19 with data augmentation.

**Table 2:** Estimation of heart rate in relaxed condition by CNNs.

Posture	Signal	MAE	SDAE	RMSE	CC
Standing	VGG-16 (No Aug)	**1.92**	**2.40**	**3.08**	0.954 **
	VGG-19 (Aug)	3.90	3.72	5.39	0.934 **
	Ensemble Network	2.07	2.56	3.29	0.960 **
Sitting	VGG-16 (No Aug)	**1.72**	**1.69**	**2.40**	0.984 **
	VGG-19 (Aug)	5.05	4.91	7.04	0.905 **
	Ensemble Network	2.52	2.50	3.55	0.970 **
Supine	VGG-16 (No Aug)	**1.67**	**2.60**	**3.09**	0.982 **
	VGG-19 (Aug)	4.06	5.04	6.47	0.946 **
	Ensemble Network	2.17	3.12	3.80	0.977 **

MAE = mean absolute error; SDAE = standard deviation of absolute error; RMSE = root mean square error; CC = Pearson’s correlation coefficient. Two asterisks represent significant correlation level at *p* < 0.01. The lowest error and highest correlation values are bolded.

**Table 3:** Estimation of heart rate in aroused condition by CNNs.

Posture	Signal	MAE	SDAE	RMSE	CC
Standing	VGG-16 (No Aug)	**2.23**	**3.92**	**4.51**	0.976 **
	VGG-19 (Aug)	7.54	7.22	10.44	0.866 **
	Ensemble Network	3.96	4.79	6.22	0.960 **
Sitting	VGG-16 (No Aug)	**2.34**	**3.47**	**4.19**	0.975 **
	VGG-19 (Aug)	8.58	9.43	12.75	0.763 **
	Ensemble Network	4.63	5.45	7.15	0.946 **
Supine	VGG-16 (No Aug)	**1.51**	**1.57**	**2.18**	0.992 **
	VGG-19 (Aug)	4.48	3.22	5.52	0.962 **
	Ensemble Network	2.28	1.81	2.91	0.988 **

MAE = mean absolute error; SDAE = standard deviation of absolute error; RMSE = root mean square error; CC = Pearson’s correlation coefficient. Two asterisks represent significant correlation level at *p* < 0.01. The lowest error and highest correlation values are bolded.

**Table 4:** Estimation of heart rate for walking by CNNs.

Speed	Signal	MAE	SDAE	RMSE	CC
3.2 km/h	VGG-16 (No Aug)	5.11	5.52	7.52	0.906 **
	VGG-19 (Aug)	7.81	6.65	10.26	0.899 **
	Ensemble Network	**5.03**	**5.29**	**7.30**	0.930 **
4.5 km/h	VGG-16 (No Aug)	5.53	6.09	8.23	0.896 **
	VGG-19 (Aug)	5.12	5.46	7.48	0.933 **
	Ensemble Network	**4.26**	**5.35**	**6.84**	0.935 **
5.8 km/h	VGG-16 (No Aug)	6.43	7.15	9.61	0.868 **
	VGG-19 (Aug)	**4.74**	**5.30**	**7.11**	0.935 **
	Ensemble Network	4.76	5.83	7.52	0.922 **

MAE = mean absolute error; SDAE = standard deviation of absolute error; RMSE = root mean square error; CC = Pearson’s correlation coefficient. Two asterisks represent significant correlation level at *p* < 0.01. The lowest error and highest correlation values are bolded.

**Table 5:** Estimation of heart rate for running by CNNs.

Speed	Signal	MAE	SDAE	RMSE	CC
6.4 km/h	VGG-16 (No Aug)	7.21	8.64	11.25	0.846 **
	VGG-19 (Aug)	**5.24**	**6.72**	**8.52**	0.917 **
	Ensemble Network	5.43	7.40	9.17	0.899 **
8.5 km/h	VGG-16 (No Aug)	8.01	8.89	11.96	0.853 **
	VGG-19 (Aug)	**5.21**	**7.14**	**8.84**	0.924 **
	Ensemble Network	5.94	7.64	9.67	0.908 **
10.3 km/h	VGG-16 (No Aug)	8.62	9.81	13.05	0.824 **
	VGG-19 (Aug)	**5.49**	**7.97**	**9.68**	0.908 **
	Ensemble Network	6.38	8.61	10.72	0.886 **

MAE = mean absolute error; SDAE = standard deviation of absolute error; RMSE = root mean square error; CC = Pearson’s correlation coefficient. Two asterisks represent significant correlation level at *p* < 0.01. The lowest error and highest correlation values are bolded.

**Table 6:** Estimation of heart rate in 12 measurement conditions by signal processing and CNN.

Condition	Method	MAE	SDAE	RMSE	CC
Sit (Relaxed)	Signal Processing	4.83	6.97	8.39	0.737 **
	CNN	**2.07**	**2.56**	**3.29**	0.960 **
Stand (Relaxed)	Signal Processing	**2.00**	**2.33**	**3.04**	0.975 **
	CNN	2.52	2.50	3.55	0.970 **
Supine (Relaxed)	Signal Processing	18.05	17.27	24.78	−0.084
	CNN	**2.17**	**3.12**	**3.80**	0.977 **
Sit (Aroused)	Signal Processing	**1.93**	**3.81**	**4.22**	0.973 **
	CNN	3.96	4.79	6.22	0.960 **
Stand (Aroused)	Signal Processing	**2.46**	**2.59**	**3.54**	0.981 **
	CNN	4.63	5.45	7.15	0.946 **
Supine (Aroused)	Signal Processing	**1.64**	**2.53**	**2.98**	0.986 **
	CNN	2.28	1.81	2.91	0.988 **
Walk (3.2 km/h)	Signal Processing	18.19	14.66	23.61	0.789 **
	CNN	**5.03**	**5.29**	**7.30**	0.930 **
Walk (4.5 km/h)	Signal Processing	14.05	12.66	19.43	0.867 **
	CNN	**4.26**	**5.35**	**6.84**	0.935 **
Walk (5.8 km/h)	Signal Processing	20.48	17.28	27.55	0.729 **
	CNN	**4.76**	**5.83**	**7.52**	0.922 **
Run (6.4 km/h)	Signal Processing	20.22	18.02	27.83	0.704 **
	CNN	**5.43**	**7.40**	**9.17**	0.899 **
Run (8.5 km/h)	Signal Processing	19.83	12.81	24.31	0.832 **
	CNN	**5.94**	**7.64**	**9.67**	0.908 **
Run (10.3 km/h)	Signal Processing	17.71	12.55	22.76	0.893 **
	CNN	**6.38**	**8.61**	**10.72**	0.886 **

MAE = mean absolute error; SDAE = standard deviation of absolute error; RMSE = root mean square error; CC = Pearson’s correlation coefficient. Two asterisks represent significant correlation level at *p* < 0.01. The lowest error and highest correlation values are bolded.
